# Epigenetic Landscape of the *Citrus* Greek National Germplasm Collection and Its Association with Genetic and Fitness-Related Data

**DOI:** 10.3390/biology15070546

**Published:** 2026-03-29

**Authors:** Fani G. Lyrou, Vasiliki Maria Kotina, Andreas G. Doulis, Nikolaos Tourvas, Vasileios Ziogas, Ioannis Ganopoulos, Filippos A. Aravanopoulos

**Affiliations:** 1 Laboratory of Forest Genetics, Faculty of Agriculture, Forestry and Natural Environment, Aristotle University of Thessaloniki, 54121 Thessaloniki, Greece; fanilyrou@for.auth.gr (F.G.L.); kotinavm@for.auth.gr (V.M.K.); tourvasn@for.auth.gr (N.T.); 2 Institute of Viticulture, Floriculture and Vegetable Crops, Hellenic Agricultural Organization DEMETER, 71003 Heraklion, Greece; doulis@elgo.iosv.gr; 3 Institute of Viticulture, Floriculture and Vegetable Crops, Hellenic Agricultural Organization DEMETER, 73134 Chania, Greece; ziogas@elgo.gr; 4 Institute of Plant Breeding and Genetic Resources, Hellenic Agricultural Organization DEMETER, 57001 Thessaloniki-Thermi, Greece; iganopoulos@elgo.gr

**Keywords:** *Citrus*, MSAP, epigenetic diversity, germplasm collection

## Abstract

Epigenetic diversity contributes to phenotypic plasticity and environmental responsiveness in *Citrus* spp. In this work, genome-wide DNA methylation was analyzed in 49 accessions representing six *Citrus* species, hybrids, varieties, and cultivars from the Greek National Germplasm Collection. Substantial variation in global DNA methylation was detected, while the epigenetic diversity indices did not differ significantly among taxa. The highest values were observed in *Citrus × aurantium* var. *sinensis* (orange) varieties (*P_epi_* = 77.33%, *Na* = 1.55, *h* = 0.14, *I_epi_* = 0.24), whereas the lowest were recorded in *Citrus × aurantifolia* (lime) (*P_epi_ =* 18.67%, *Na* = 0.37, *h =* 0.09, *I_epi_* = 0.13), reflecting potential methylation restructuring impacted by hybridization and selection. Epigenetic and genetic diversity were significantly different. Principal coordinate analyses (PCoA) of epigenetic data revealed limited concordance to taxonomy, except for unmethylated loci, the latter exhibiting similar data to genetic (SSR) results in which groups reflected the taxonomic genealogy. Epigenetic and genetic distances were uncoupled, and associations between epigenetic diversity (*P_epi_*, *h*, *I_epi_*) and traits directly or indirectly related to fitness (fruit weight, dry matter content, ascorbic acid concentration), were weak. These findings indicate that epigenetic diversity represents an independent layer of variation in *Citrus* germplasm with potential relevance for breeding, conservation and environmental resilience.

## Simple Summary

Citrus plants, such as oranges, mandarins, lemons and limes, show widespread differences in fruit quality and their ability to adapt to environmental conditions, but it is not fully understood how much of this variation is influenced by epigenetics, which involves chemical changes to DNA that affect gene activity without altering the genetic code itself. This study examined DNA methylation, a key epigenetic mechanism, in 49 known citrus varieties from the Greek National Germplasm Collection. The aim was to find out how much epigenetic diversity is present, to compare it with genetic diversity, and to explore whether it is associated with important fruit characteristics. We found considerable variation in DNA methylation among citrus varieties, with sweet orange varieties showing the highest levels of epigenetic diversity and lime showing the lowest. Importantly, epigenetic diversity differed significantly from genetic diversity, showing that it represents a separate layer of biological variation. However, the relationship between epigenetic diversity and traits, such as fruit weight and vitamin C levels, was weak. Overall, the findings suggest that epigenetic variation provides an additional resource for citrus breeding, conservation, and the development of varieties that are better adapted to environmental challenges and climate change, benefiting agriculture and society.

## 1. Introduction

*Citrus* spp. (*Citrus* L., Rutaceae) are amongst the most important fruit crops worldwide, bearing significant agricultural and commercial importance [1,2]. Main citrus-producing areas cover the Mediterranean basin, South-East Asia, Eastern Australia, Japan, South Africa, Madagascar, and the Americas [3]. Commercially, several *Citrus* fruits, such as bergamot, lemon, lime, mandarin and orange, are widely accepted by consumers globally, due to their appearance, flavors and appealing aromas [4,5]. Conventional breeding has a generally limited impact on citrus genetic improvement, due to the complex taxonomic relationships among cultivar groups [2,6]. Most citrus cultivars are interspecific hybrids, with genomes composed of large DNA fragments inherited from basic taxa. The individual roles of the ancestral taxa in shaping the genomic architecture of certain secondary cultivated species are becoming increasingly well-defined [7]. Over the past two decades, research has expanded on epigenetic changes in plants, more specifically on DNA methylation status, and on how environmental factors (abiotic stresses, pedoclimatic conditions, climate change) influence their responses and adaptation. Since genetic markers alone may underestimate functional diversity, epigenetic profiling can reveal hidden variation that is relevant to fitness. Although *Citrus* species often exhibit low genetic diversity due to domestication effects and extensive clonal propagation, this genetic stability is frequently decoupled from the considerable levels of phenotypic and metabolic diversity observed in germplasm collections [5,8,9]. To uncover this gap, implementing an epigenetic profiling approach is critical to address a variation that is potentially independent of DNA sequence changes and connects the adaptive plasticity and fitness-related traits [10,11,12]. Therefore, germplasm collections may harbor substantial epigenetic resources, even when their genetic basis is restricted. Most epigenetic studies on plants have insofar employed the AFLP-based Methylation-Sensitive Amplified Polymorphism (MSAP) analysis. This method involves two parallel double enzymatic digestions of DNA, using EcoRI in combination with two isoschizomers with different methylation sensitivities: HpaII and MspI. Evaluating the two MSAP profiles reveals the samples’ DNA methylation status [13]. The method has been popular, especially for non-model species, due to its low cost.

Gene regulation through genetic and epigenetic processes is essential for phenotyping in eukaryotic organisms [12,14,15,16,17,18]. This especially occurs in grafted Citrus species, where somatic and epigenetic mutations can be systematically transmitted across the graft plant and maintained through clonal propagation [19]. Such modifications drive phenotypic diversity and stress resilience without requiring changes to the underlying DNA sequence [20,21]. Epigenetic modifications include DNA methylation [22], histone changes [23], histone variants and small RNAs [24], which can show transgenerational inheritance. Continuous environmental changes underscore the need for a more extensive study of epigenetic variability. Moreover, including epigenetic variability in breeding programs is of high importance [12,18]. The first study on DNA methylation in citrus plants was conducted by Neves et al. [20]. They studied how repeated drought exposure affects the epigenetic profiles, which may be responsible for phenotypic variation, including agronomic traits of high interest, such as drought tolerance. However, this study only focused on a limited number of genotypes, and further studies are needed to expand the genetic base of the genus *Citrus* [19].

A major *Citrus* germplasm collection is maintained at the ex situ Fruit Tree Collection of the Hellenic Agricultural Organization ELGO-DEMETER, located in Chania, Greece. This collection consists of 224 accessions with diverse phenotypic traits and geographic origins. It includes both international and Greek accessions, many of which have yet to be thoroughly characterized at the genetic and epigenetic levels [25]. The collection includes 49 accessions (3 bergamots, 3 citrons, 2 limes, 12 lemons, 10 mandarins, and 19 oranges) from different regions, along with commercial accessions from the UC Riverside Citrus Variety Collection, Riverside, California (USA) and INRAE (Corsica, France). The Greek local accessions were assembled through targeted expeditions, with the aim of preserving *Citrus* genetic resources at the national level and securing germplasm for future breeding. Some accessions, such as the common citron, have been cultivated in Greece for nearly 2000 years, whereas others, such as the well-known ‘Chios mandarin’, have a recorded history of about 500 years. Most of these accessions are indigenous landraces, traditionally used in Greek agriculture since the early 20th century. In recent years, the collection has been expanded to include commercially available varieties sourced from regional nurseries [26].

Herein, we used the MSAP approach to assess the epigenetic diversity of 49 accessions from six *Citrus* taxa present at the ex situ Greek National Germplasm Collection. We studied the variation in DNA methylation patterns and investigated potential correlations with species’ identity, taxonomic relationships and phenotypic traits related to fitness. With the combination of epigenetic, as well as published genetic and quantitative trait data, the present study contributes to a better understanding of the role of epigenetic variation in the adaptation of *Citrus* spp. under emerging novel stresses linked to climatic change. This epigenetic plasticity is of particular importance in *Citrus*, where narrow genetic bases, resulting from clonal propagation, may reduce the adaptive responses. By providing a mechanism for rapid phenotypic plasticity through the regulation of gene expression, a swift integration of environmental signals into stable physiological adjustments can be achieved, enhancing the capacity of perennial crops to adapt to the accelerated climatic changes [27].

## 2. Materials and Methods

### 2.1. Plant Material and DNA Extraction

The 49 *Citrus* accessions that were present in the ex situ ELGO-DEMETER germplasm collection were sampled. These accessions were classified under the following species [8]: *C. × limon* var. *bergamia* (bergamot), *C. × medica* (citron), *C. × aurantifolia* (lime), *C. × limon* var. *limon* (lemon), *C. × reticulata* (mandarin) and *C. × aurantium* var. *sinensis* (orange) (Table A1). Young leaves from each accession were collected, rapidly frozen in dry ice, and stored at −80 °C. For DNA extraction, leaves were ground into a fine powder, using beads in a Precellys^®^ homogenizer (Bertin Technologies, Montigny-le-Bretonneux, France). DNA extraction was performed using the NucleoSpin Plant II Kit (Macherey-Nagel, Düren, Germany), according to the manufacturer’s protocol. All DNA samples were diluted to 20 ng/μL and then stored at −20 °C for further analysis.

### 2.2. MSAP Analysis

MSAP (Methylation-Sensitive Amplified Polymorphism) is a modified version of the common AFLP technique. In the MSAP method, EcoRI acts as the rare cutting enzyme, while the methylation-sensitive restriction enzymes HpaII and MspI work as frequent cutters. These two enzymes are isoschizomers and they recognize the same target sequence, 5′-CCGG-3′. However, they are extremely sensitive to methylation, depending on whether it takes place at the internal or external cytosine. For the MSAP analysis, the total genomic DNA per reaction was 300 ng and was digested with 4 U of EcoRI (New England Biolabs, NEB, Ipswich, MA, USA) and 4 U of HpaII (New England Biolabs), and 300 ng was subjected to 4 U of EcoRI and 4U of MspI (New England Biolabs). The procedure of digestion was carried out at 37 °C for 1 h and 65 °C for 20 min (heat inactivation). The digested DNA fragments and the EcoRI and HpaII/MspI adapters (Table 1) were ligated at 25 °C for 2 h, with the use of 400 U/μL of T4 DNA ligase (New England Biolabs). After the incubation period, the samples were treated to a heat shock reaction for 10 min at 65 °C to end the ligation step. A set of primers based on the sequences of the EcoRI and HpaII/MspI adapters (Table 1), specifically EcoRI + A, HpaII/MspI + A or HpaII/MspI + T, was used for the pre-selective PCR reaction. Pre-amplification PCR was conducted in a total volume of 20 μL, including 10× Kapa Taq Buffer; 10 mM dNTPs; 50 ng of each primer EcoRI + A, HpaII/MspI + A or HpaII/MspI + T, 5 U Taq DNA polymerase (2G, Robust, Hotstart, KK5005, Kapa Biosystems, Roche, Basel, Switzerland); and 5 μL of diluted fragments (products from digestion and ligation reactions). Cycling was carried out on a SimpliAmp™ Thermal Cycler (Applied Biosystems™, ThermoFisher Scientific, Waltham, MA, USA) with a 95 °C hold for 4 min, followed by 23 cycles of 95 °C for 30 s, 56 °C for 60 s and 72 °C for 2 min and afterwards followed by a final hold at 72 °C for 2 min. To verify the amplification, a 5 μL aliquot of the pre-selective PCR step was electrophoresed on agarose; the remaining 15 μL were diluted 10-fold with TE. A selective amplification PCR was performed in 20 μL total volume, consisting of 10× Kapa Taq DNA polymerase, 10 mM dNTPs, 0.1 μL of each EcoRI primer (50 ng/μL), 0.6 μL of HpaII/MspI primer (50 ng/μL), 5 U Taq DNA polymerase (2G, Robust, Hotstart, KK5005, Kapa Biosystems), 3 μL of diluted pre-selective product and distilled water up to the final volume. Cycling was carried out on a SimpliAmp™ Thermal Cycler (Applied Biosystems™, ThermoFisher Scientific) with a 95 °C hold for 4 min, followed by 1 cycle of 95 °C for 30 s, 65 °C for 30 s and 72 °C for 1 min, subsequently followed by 12 cycles (−1 °C each, touchdown) of 95 °C for 30 s, 65 °C for 30 s and 72 °C for 1 min, afterwards followed by 23 cycles of 95 °C for 30 s, 56 °C for 30 s and 72 °C for 1 min, with a final hold at 72 °C for 1 min.

### 2.3. Scoring of MSAP Markers

The size of the detected fragments was scored in GeneMapper v6.0. Stable fragments in the range of 100 to 500 bases were included and analyzed deeper to avoid the impact of potential size homoplasy [28]. The analysis of the MSAP results included a comparison of the EcoRI/HpaII and EcoRI/MspI reaction combinations, which were analyzed using the MSAP_calc.r script in RStudio (v2024.12.1+563, Posit Software, Boston, MA, USA), leading to the classification into four categories of a specific fragment: A—fragments present in both profiles (1/1), indicating an unmethylated state; B—fragments present only in EcoRI/MspI profiles (0/1), indicating semi- or fully methylated CG sites; C—fragments present only in EcoRI/HpaII profiles (1/0), indicating semi-methylated CHG sites; and D—absence of fragments in both profiles (0/0), representing a non-informative state caused either by different types of methylation or due to restriction site polymorphism [18,29,30].

### 2.4. Comparative Analysis Between Epigenetic and Genetic Data

Epigenetic diversity statistics within taxa were analyzed using GenAlEx 6.502b [31,32]. The calculated parameters included: the percentage of polymorphic loci (*P_epi_*), number of alleles (*N_a_*), effective number of alleles (*A_e_*), diversity (*h*), non-biased diversity (*uh*) and Shannon’s Diversity Index (*I_epi_*). To conduct a comparative analysis between the genetic and epigenetic data, we have calculated genetic diversity parameters for the 49 accessions studied using the data of Tourvas et al. [26] (14 microsatellite loci). Genetic analysis was performed in GenAlEx 6.502b and the following genetic diversity parameters were calculated: percentage of polymorphic loci (*P*), number of alleles (*N_a_*), effective number of alleles (*A_e_*), observed heterozygosity (*H_o_*), expected heterozygosity (*H_e_*) and Shannon’s Diversity Index (*I*). For the latter parameter, statistically significant differences could be investigated, and this was carried out using a Mann–Whitney test/Concordance, and genetic and epigenetic distances were tested using the Mantel Test. Principal Coordinate Analysis (PCoA) was used to assess differentiation among taxa, based on the genetic (SSR) and epigenetic (MSAP) data. The same analysis was carried out to assess differentiation between accessions within taxa for the three taxa with an adequate sample size: lemon, mandarin and orange (Table A1). Regarding epigenetic analysis, PCoAs were also conducted after partitioning the epigenetic dataset according to the different subepilocus methylation types: unmethylated (u), CHG-hemimethylated (h) and CG-methylated. Principal Coordinate Analyses (PCoAs) were used to assess the differentiation among accessions within subtaxa that were represented by sufficient sample sizes, *C. × limon* var. *limon* (lemon), *C. × reticulata* (mandarin), and *C. × aurantium* var. *sinensis* (orange). In addition, epigenetic PCoAs were carried out seperately for each subtaxon after the separation of the MSAP dataset according to subepilocus methylation status, including unmethylated (u), CHG-hemimethylated (h), and CG-methylated loci, in order to examine the distribution of accessions of each subataxa along the principal coordinate axes.

Pairwise *t*-tests were employed to assess differences in unbiased diversity (*h*) and Shannon’s Diversity Index (*I_epi_*) among *C. × limon* var. *limon*, *C. × reticulata*, and *C. × aurantium* var. sinensis. Analyses were only performed for taxa represented by adequate sample sizes to ensure statistical robustness. Statistical significance was determined at a threshold of *p* < 0.05. All analyses were performed in the R statistical environment.

### 2.5. Associations of Epigenetic Data with Fitness Surrogate Traits

Associations of traits related to fitness with epigenetic diversity were investigated to explore the potential links between the molecular and phenotypic differences among Citrus taxa. All accessions included in the MSAP analysis were also evaluated for three quantitative traits related to the fitness of the endocarp fruit weight (g), dry matter content (%), and ascorbic acid concentration (mg/100 mL) [5]. The former is directly and the two latter are indirectly related to fitness [33,34,35]. Correlation analyses were performed in RStudio between these traits and the epigenetic diversity parameters: epigenetic diversity (*h*), polymorphism rate (*P*), and Shannon’s Diversity Index (*I*). This analysis aimed to examine the potential functional relationships between epigenetic variation and fitness–associated traits.

## 3. Results

### 3.1. Epigenetic Diversity Analysis

A total of 75 stable and repeatable epiloci (31 methylated, 7 unmethylated, 37 hemimethylated), were detected in all accessions. Among the taxa, orange exhibited the highest epigenetic diversity (Table 2), in terms of the number of different alleles (*N_a_* = 1.55), percent of polymorphic loci (*P_epi_* = 77.33%), diversity (*h* = 0.14) and Shannon’s Diversity Index *I_epi_* = 0.24). Lemon (*P_epi_* = 53.33%) and mandarin (*P_epi_* = 46.67%) followed. Lime exhibited the lowest values (*N_a_* = 0.37, *P_epi_* = 18.67%, *h* = 0.09, *I_epi_* = 0.13). Epigenetic diversity values did not differ markedly among taxa, showing a moderate and broadly conserved degree of methylation pattern.

### 3.2. Genetic Diversity Analysis

A total of 70 stable and repeatable loci were detected in all species. Genetic diversity was higher in mandarin (*N_a_* = 1.55, *p* = 92.86%) and bergamot (*I* = 0.71, *H_o_* = 0.67), whereas orange displayed the lowest genetic diversity across several parameters, including Shannon’s Diversity Index (*I* = 0.35) and expected heterozygosity (*H_e_* = 0.24) (Table 3).

The analysis (Table 2 and Table 3) reveals a contrasting trend between genetic and epigenetic diversity profiles in certain *Citrus* taxa within the collection. While orange exhibited the lowest genetic diversity (*I* = 0.35, *P%* = 71.43%), it maintained the highest epigenetic diversity (*P_epi_* = 77.33%, *h* = 0.14, *I_epi_* = 0.24). On the other hand, mandarin presented the highest percentage of genetic polymorphism (*P%* = 92.86%), but its epigenetic polymorphism (*P_epi_* = 46.67%) was comparatively lower. This suggests that for taxa with a narrow genetic base, such as orange, epigenetic variation may play a more important role in providing the phenotypic plasticity that is necessary for adaptation. Lime consistently showed low diversity in both analyses, ranking lowest in epigenetic polymorphism (*P_epi_* = 18.67%) and having a small number of alleles (*N_a_* = 2.00).

The pairwise *t*-tests conducted to compare unbiased epigenetic diversity (*h*) and Shannon’s Diversity Index (*I*) were employed in *C. × limon* var. *limon* (lemon), *C. × reticulata* (mandarin), and *C. × aurantium* var. *sinensis* (orange) (Table 4). No statistically significant differences were observed. These results indicate that despite differences in polymorphism percentages, the overall epigenetic diversity levels are statistically comparable across these three species, suggesting that accessions in the germplasm collection maintain similar patterns of epigenetic variation.

The overall genetic and epigenetic diversity values based on Shannon’s Diversity Index for the collection were compared using a Mann–Whitney test. The difference between the two analyses was found to be statistically significant (*p* = 0.031).

### 3.3. Comparison of Epigenetic and Genetic Diversity

**Figure 1:**
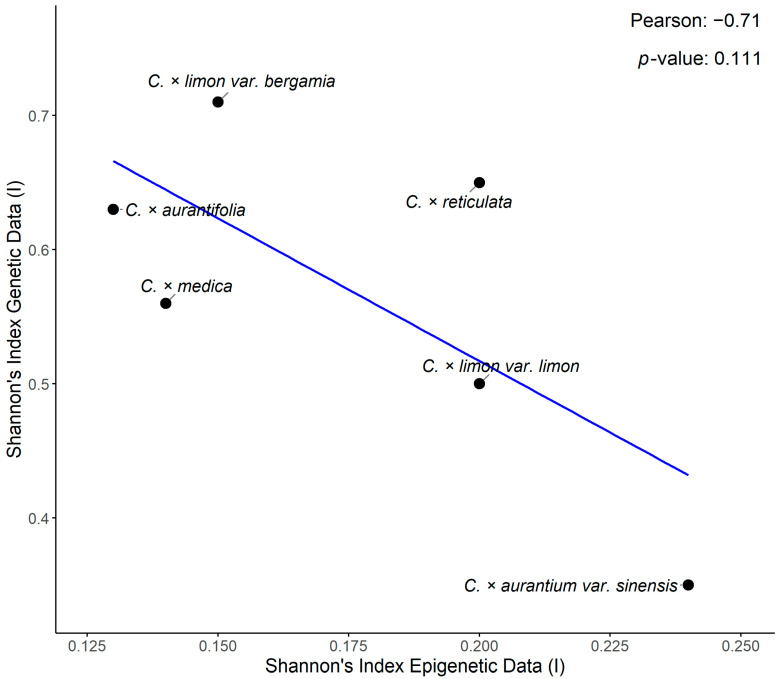
Pearson’s correlation analysis of the Shannon’s Diversity Index (I) of genetic and epigenetic data of six accessions of *Citrus* spp.

Polymorphism based on genetic markers was greater in all species, with the exception of orange, where epi-polymorphism was slightly higher (Table 2 and Table 3). Diversity, as indicated by the common parameter of the Shannon’s Diversity Index, was also higher when estimated using genetic markers across all six taxa (Table 2 and Table 3, Figure 1). Pearson’s correlation analysis of genetic and epigenetic Shannon’s Diversity Index values revealed a strong but statistically non-significant (r = −0.71, *p* = 0.11) negative correlation between the six taxa studied (Figure 1).

**Figure 2:**
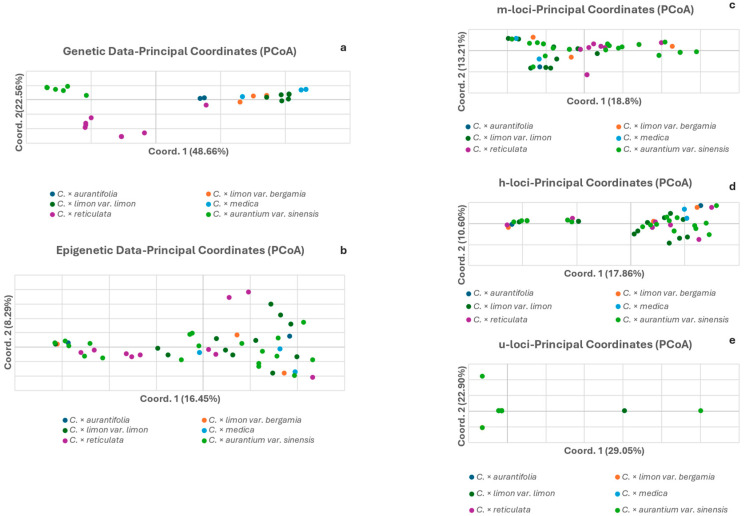
Principal coordinate analyses (PCoA) of genetic (**a**) and epigenetic distances (**b**) of *Citrus* spp. Epigenetic data were further partitioned into three methylation types: (**c**) CG-methylated and (**d**) CHG-hemi-methylated; (h) subepiloci and (**e**) unmethylated; and (u) subepiloci.

In the genetic data PCoA, a large part of the total variation is explained by the first two axes (48.66% and 22.56%, respectively, Figure 2a), showing clear clustering patterns that correspond to the underlying taxa. Specifically, three distinct patterns were observed: the *C. × limon* and *C. × medica* group, the *C. × aurantifolia* and *C. × reticulata* group, and the *C. × aurantium* group. In contrast, the epigenetic data PCoA explained roughly 3× less of the total variation in the first two components (16.45% and 8.29%, respectively, Figure 2b). Furthermore, no clustering was evident among the taxa, either in terms of overall methylation patterns or in specific methylation analysis categories (Figure 2c–e). The PCoA of CG-methylated types (m-loci) (Figure 2c) did not display groupings, something also seen in Figure 2d, which displays CHG-hemimethylated types (h) subepiloci, and in Figure 2e, which shows CHH-unmethylated types (u-loci). In the PCoA of CHH-unmethylated types (u-loci) (Figure 2e), a significant number of accessions from different taxa overlap at a single point near the center of the *x*-axis. This clustering indicates a lack of epigenetic differentiation among these taxa at the CHH-unmethylated level, suggesting that these specific u-loci do not vary across most of the germplasm collection.

**Figure 3:**
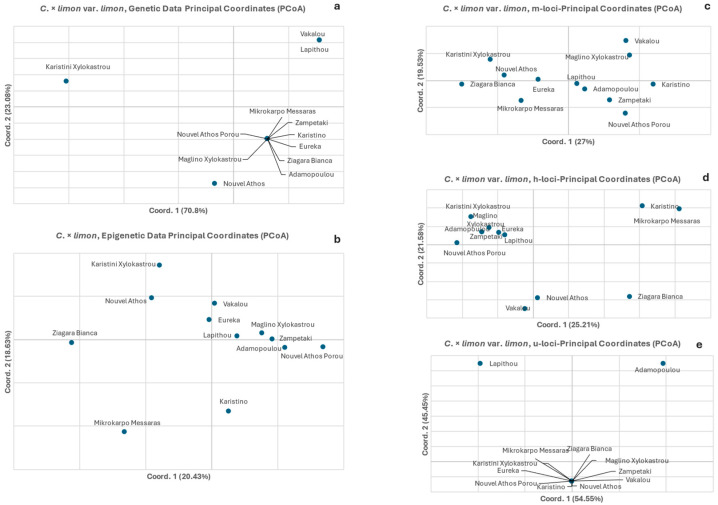
Principal coordinate analyses (PCoA) for *C*. *× limon* var. *limon* (lemon) of genetic (**a**) and epigenetic distances (**b**) of *Citrus* spp. Epigenetic data were further partitioned into three methylation types: (**c**) CG-methylated, (**d**) CHG-hemimethylated (h) subepiloci, and (**e**) unmethylated (u).

The PCoA of the *C. × limon* var. *limon* (lemon) accessions genetic data resolved almost all the variation in the two-dimensional multivariate space (70.80% on the 1st component and 23.08% on the 2nd, Figure 3a). The PCoA depicted all accessions but three (‘Karystini Xylokastrou’, ‘Vakalou’ and ‘Lapithou’, which represent different Greek lemon landraces) in a single group. The respective analysis of the epigenetic data (Figure 3b) resolved almost 2.5× less variation (39.06% in the first two components), while no grouping was observed. Figure 3c, which represents CG-methylated types (m-loci), explained 46.53% of the total variation in the first two components with no clear grouping as well. Figure 3d, representing CHG-hemimethylated (h) subepiloci types, explains a variation that is similar to the previous amount of the total variation (46.79% in the first two axes), while one group including 58% of the lemon accessions is seen. In Figure 3e, which displays unmethylated types (u-loci), all variation is explained in the first two axes, while all accessions but two form a single group.

**Figure 4:**
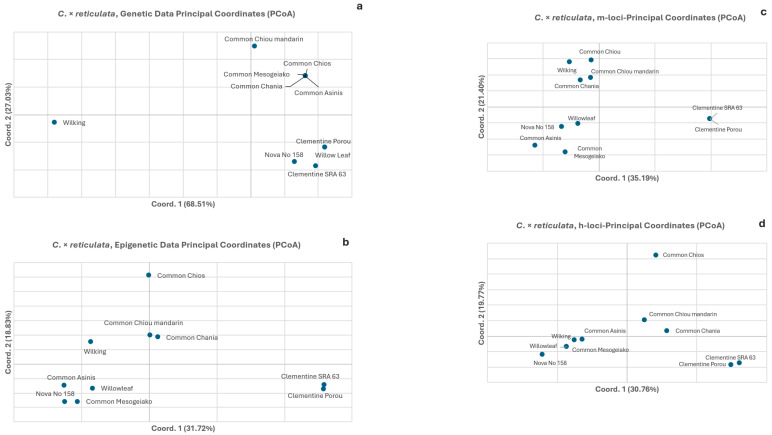
Principal coordinate analyses (PCoA) for *C. × reticulata* (mandarin) of genetic (**a**) and epigenetic distances (**b**) of *Citrus* spp. Epigenetic data were partitioned into three methylation types: (**c**) CG-methylated and (**d**) CHG-hemimethylated (h) subepiloci.

The mandarin PCoA of the genetic data (Figure 4a) also explained almost all the total genetic variation (68.51% in the 1st axis and 27.03% in the 2nd). All accessions but one (‘Wilking’, the result of a cross between the hybrid ‘Tangor’ (*C. × reticulata × C. × sinensis*) and the common ‘Mediterranean mandarin’ (*C. × reticulata*), i.e., containing a part of the orange genome) form a loose group. These results are consistent with the origin of the accessions. The clear separation of ‘Wilking’ from the other mandarin accessions along the first coordinate (68.51%) highlights its distinct genetic profile, due to its hybrid ancestry. In contrast, the PCoA of the epigenetic data (Figure 4b) explained 2× less of the total (50.55%), while one loose group (with a different composition from the genetic data PCoA’s group) was depicted. The PCoA of the CG-methylated and CHG-hemimethylated subepiloci (Figure 4c,d) explained a similar amount of the total variation (56.59% and 40.53% respectively), and showed a similar accession orientation type, forming one loose group (more pronounced in the CG-methylated type). It is worth noting that the two clementines (Clementine Porou and Clementine SRA 63) were not included in any group, either in the genetic or in the different epigenetic multivariate analyses. Finally, the PCoA for CHH-unmethylated subepiloci (u-loci) was not performed, as no unmethylated u-loci were detected among the *C. × reticulata* (mandarin) accessions during the analysis. The epigenetic structure was primarily determined by the distribution of the CG-methylated and CHG-hemimethylated subepiloci.

**Figure 5:**
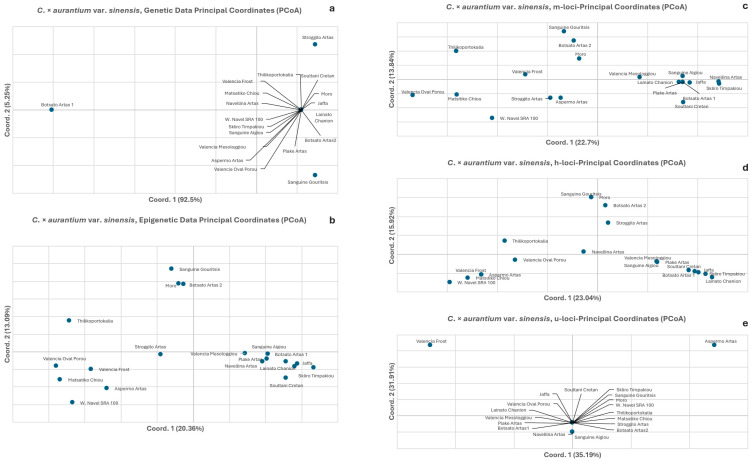
Principal coordinate analyses (PCoA) for *C*. *× aurantium* var. *sinensis* (orange) of genetic (**a**) and epigenetic distances (**b**) of *Citrus* spp. Epigenetic data were further partitioned into three methylation types: (**c**) CG-methylated, (**d**) CHG-hemimethylated (h) subepiloci, and (**e**) unmethylated (u).

Regarding the analysis of *C. × aurantium* var. *sinensis* (orange) accessions, the PCoA based on genetic data (Figure 5a) explained almost all the total variation: 92.50% in the first axis and 5.25% in the second. Most accessions are grouped together, except for ‘Botsato Artas 1′ (and to a lesser extent, ‘Stroggilo Artas’ and ‘Sanguine Gouritsis’). The PCoA of the epigenetic data (Figure 5b) resolves almost 3× less variation in 2D multivariate space (33.45%), whilst exhibiting three loosely formed groups. A low amount of variation was seen (Figure 5c,d) in the PCoAs of the m-loci (CG-methylated) and h-loci (CHG-hemimethylated) subepiloci (36.54% and 38.96%, respectively), while a loose grouping similar to that above was observed. The PCoA of the unmethylated type (Figure 5e) explained a higher than before percentage of the total variation (67.10%) and depicted one large group, similar to the result of the genetic data PCoA.

In the PCoA analyses of epigenetic distances for *C. × limon* var. *limon* (lemon), *C. × reticulata* (mandarin), *C. × aurantium* var. *sinensis* (orange) (Figure 3, Figure 4 and Figure 5), no distinct grouping of accessions within species was observed, either in terms of overall methylation patterns or within specific methylation categories (CG-methylated, CHG-hemimethylated, unmethylated). In contrast, the PCoA based on genetic distances revealed clear grouping of most accessions in all three taxa.

The results of the Mantel tests to assess genetic–epigenetic distance correlations and to determine whether genetic and epigenetic divergence is concordant, were performed across all *Citrus* spp. accessions, and within the taxa that encompass sufficient sample size for this analysis (*C. × limon* var. *limon*, *C. × reticulata* and *C. × aurantium* var. *sinensis*; Table 5). The Mantel tests indicated no significant correlation between genetic and epigenetic distances (R^2^ range 0.0002–0.0542, *p*-values range 0.160–0.360).

### 3.4. Associations of Epigenetic Data with Fitness Surrogate Traits

**Figure 6:**
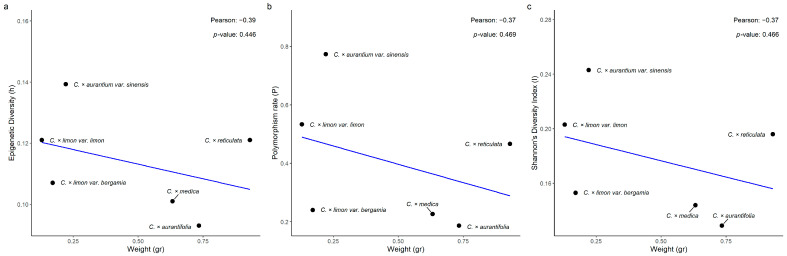
Pearson’s correlation analysis of (**a**) epigenetic diversity (h), (**b**) polymorphism rate (P), and (**c**) Shannon’s Diversity Index (I), with endocarp’s weight (gr), across six *Citrus* spp. taxa.

**Figure 7:**
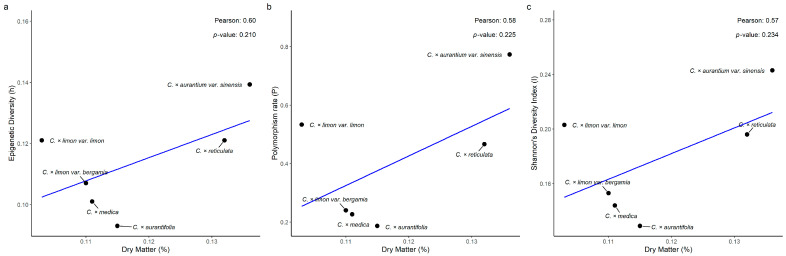
Pearson’s correlation analysis of (**a**) epigenetic diversity (h), (**b**) polymorphism rate (P), and (**c**) Shannon’s Diversity Index (I), with endocarp’s dry matter (%), across six *Citrus* spp. taxa.

Pearson’s correlation analysis of the epigenetic diversity (h) and endocarp’s weight data revealed a weak negative and not statistically significant correlation across the six taxa (r = −0.39, *p* = 0.447; Figure 6a). Furthermore, a moderate negative, not statistically significant, correlation was observed between the polymorphism data (P) and the endocarp’s weight (Figure 6b; r = −0.37, *p* = 0.469). The correlation between Shannon’s Diversity Index and the endocarp’s weight was moderately negative and not statistically significant (r = −0.37, *p* = 0.466; Figure 6c). Pearson’s correlation analysis of the epigenetic diversity (h) and endocarp’s dry matter revealed a weak positive and not statistically significant correlation across the six taxa (r = 0.60, *p*-value = 0.211; Figure 7a). Moreover, a moderate positive, though not statistically significant, correlation was observed between polymorphism data (P) and endocarp’s dry matter (r = 0.58, *p* = 0.225; Figure 7b). Finally, the correlation analysis between Shannon’s Diversity Index and the endocarp’s dry matter exhibited a moderate positive and not statistically significant correlation (r = 0.57, *p* = 0.234; Figure 7c).

**Figure 8:**
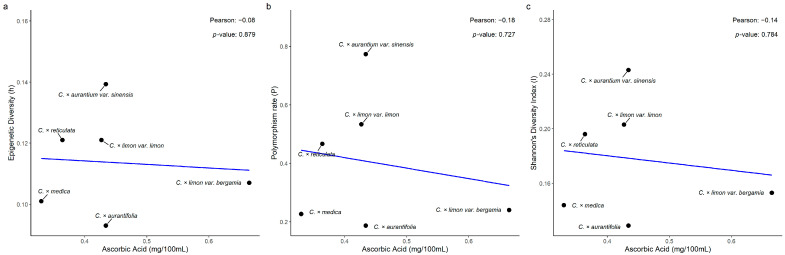
Pearson’s correlation analysis of (**a**) epigenetic diversity (h), (**b**) polymorphism rate (P), and (**c**) Shannon’s Diversity Index (I), with endocarp’s ascorbic acid (mg/100 mL), across six *Citrus* spp. taxa.

The correlation of epigenetic diversity (h) and the endocarp’s ascorbic acid was weakly negative and not statistically significant across the six taxa studied (r = −0.08, *p* = 0.879; Figure 8a). A weak negative and not statistically significant correlation was also observed between the polymorphism data (P) and the endocarp’s ascorbic acid (r = −0.18, *p*-value = 0.727; Figure 8b). Finally, the correlation between Shannon’s Diversity Index and the endocarp’s ascorbic acid content was weakly negative and not statistically significant (r = −0.14, *p* = 0.784; Figure 8c).

## 4. Discussion

The principal aim of the present study was to reveal the extent and structure of the global epigenetic diversity of the Greek ex situ national *Citrus* collection. Epigenetic mechanisms, particularly DNA methylation, may play a crucial role in the adaptation and plasticity of citrus to various environmental conditions [20] while providing a mechanism for rapid phenotypic adjustments [36].

Overall, the results have shown a wealth of epigenetic diversity, both across the collection and within taxa. This is attested by the comparative analysis of the relevant literature, which regrettably is very limited. For instance, by studying 18 clementine cultivars, ref. [37] found an average MSAP *P_epi_* = 8% compared to *P_epi_* = 46.7% found in mandarins in the present study (Table 2), while by studying two Valencia orange cultivars, ref. [20] found an average MSAP *P_epi_* = 60.25% compared to *P_epi_* = 77.30% found in oranges in the present study (Table 2). Avramidou et al. [18] found MSAP epigenetic diversity in 22 sweet cherry cultivars that exhibited lower polymorphism, but higher epigenetic diversity (*P_epi_* = 66.08, h*_epi_* = 0.20, *I_epi_* = 0.32; compared to *P_epi_* = 40.44, h = 0.11, *I_epi_* = 0.18; Table 2), which was comparable to our results.

The comparison of epigenetic and genetic diversity within the ex situ germplasm collection revealed important taxa differences. For instance, while orange accessions exhibited the highest epigenetic diversity, lemon and mandarin displayed the highest genetic diversity, suggesting distinct evolutionary and domestication pressures acting on these two types [7]. The high epigenetic diversity in oranges might be attributed to environmental interactions and horticultural practices that induce methylation changes, whereas the pronounced genetic diversity in lemons and mandarins could stem from their extensive hybridization history and broad geographic distribution [7]. The above results are concordant to findings in other plant species [18,36].

At the global level, epigenetic diversity was significantly lower than genetic diversity (*I_epi_* = 0.18, *I* = 0.57). Epigenetic and genetic diversity appear to be essentially decoupled. This result was also detected in *Citrus paradisi*, where epigenetic, but not genetic, changes were detected [38]. In *Citrus*, genetic and epigenetic diversity are found to be decoupled, probably due to their long lifespan, clonal propagation and grafting. The above allow for mitotically heritable DNA methylation epimutations to accumulate at much higher rates than DNA mutations, persisting across clonal generations, and generating substantial epigenetic diversity, even within genetically uniform cultivars [39,40]. Grafting and environmental stresses further amplify this decoupling [20,41]. Decupling between epigenetic and genetic diversity has been shown in other species: for instance, in *Prunus*, likely being linked to domestication [18,42]; in *Acer rubrum* and *Pinus nigra*, potentially linked to pollution [30,43]; and rapid environmental adaptation [44], indicating distinct evolutionary pathways [10].

The comparative analyses of the epigenetic and genetic data as depicted in the PCoAs clearly show that while the genetic results are concordant to taxa phylogeny and taxonomic genealogy, this is not reflected in the epigenetic results (Figure 3a,b, Figure 4a,b and Figure 5a,b). The absence of particular groupings is extended, besides the global methylation PCoAs, to the PCoAs of the sub-epiloci methylation classes (Figure 3c–e, Figure 4c–d and Figure 5c–e). The PCoAs of epigenetic distances for lemon, mandarin and orange (Figure 3, Figure 4 and Figure 5) revealed no distinct grouping of within-species accessions, both in overall methylation and within methylation categories. In contrast, PCoA based on genetic distances revealed a clear grouping of most accessions in all taxa, reflecting the close genetic relationships and phylogenetic similarity of the underlying genetic material, with only a small number of accessions exhibiting differentiation. There is a clear emerging pattern: the genetic data depict a specific orientation of the accessions in the formation of one major group, which is absent in the epigenetic analysis (total methylation and CG-methylation analysis). However, this grouping is restructured to a significant extent in the hemimethylated subepiloci analysis, though to a limited degree, and is almost fully reformed in the unmethylated subepiloci analysis. It appears that DNA methylation dynamics completely alter the accession orientation in the low multivariate space created by the genetic data in all taxa, which is somewhat restored when the hemi-methylated and especially the un-methylated data sets are used. These results highlight a divergence between the genetic and epigenetic clustering patterns in multivariate space, confirming that genetic similarity does not dictate epigenetic profiles in the taxa studied.

Global genetic and epigenetic diversity (*I_epi_*) were significantly different, specifying that the levels of epigenetic and genetic diversity are not concordant across all accessions. Moreover, the Mantel tests indicated no significant correlation between genetic and epigenetic distances, a finding supported by the Pearson’s correlation analysis of the Shannon’s Diversity Index values (r = −0.71, *p*-value = 0.11; Figure 1). The lack of correlation suggests that epigenetic diversity in these taxa is not strictly connected to genetic structure. The absence of species grouping in the epigenetic PCoAs, combined with the non-significant Mantel results, verify that epigenetic variation is clearly decoupled from genetic diversity in the studied *Citrus* taxa.

The relationships of epigenetic diversity (*P_epi_*, *h*, *I_epi_*) to traits that are directly or indirectly related to fitness (endocarp fruit weight, dry matter content, ascorbic acid concentration) were also investigated. Nevertheless, none of the nine correlations examined proved to be high or statistically significant. The developmental pathways regulating these quantitative traits (cell division, expansion, and sugar-water accumulation for endocarp fruit weight; carbohydrate accumulation, cell wall composition, and water balance for dry matter content; biosynthesis, recycling, and degradation pathways for endocarp ascorbic acid content) are sensitive to the epigenetic regulation of fruit development and metabolism [45].

The potential of epigenetic variation for the influence in the fitness-related traits in *Citrus* is significant, given the long-lived nature of these woody perennial species. Epigenetic modifications, such as DNA methylation, act as a mechanism for phenotypic plasticity, allowing plants to adapt in environmental change without permanent genetic changes [11,46]. In the six taxa of the present study, this functional relationship is particularly evident during stress responses, such as water deficits and ABA sensitivity, potentially enhancing survival across extreme drought events [19,20]. Epimutations can offer a stable memory of past stresses, providing a mechanism for rapid adaptation that is critical for the resilience of clonal germplasm collections [21,47].

However, the lack of significant correlations between epigenetic markers and specific traits observed in this study is potentially a consequence of the use of the MSAP technique. MSAP is a rather low-resolution profiling method, effectively ignoring methylation in CHH and CHG contexts that are significant for gene regulation [48,49]. Furthermore, MSAP markers exhibit an unclear functional link to specific genes or regulatory elements, which complicates the interpretation of how epigenetic diversity can be reflected in phenotypic outcomes [46,50]. This often results in an unknown relationship between genetic and epigenetic profiles, where the two seem to be decoupled despite their shared connection in defining plants’ phenotypes [47,51]. Nevertheless, MSAPs remain a first-pass technique of choice for an initial low-level screening, as a low-cost, limited resolution screening tool [36], compared to bisulfite sequencing, a high-resolution, sequence-specific method. Overall, the absence of significant correlations found in this study is likely the result of the MSAP resolution level and points towards the future use of epigenomic profiling in such studies.

## 5. Conclusions

The results of this study demonstrate a wealth of epigenetic diversity in the Greek ex situ national *Citrus* accessions and notable epigenetic diversity within taxa, while the epigenetic diversity parameters do not differ significantly among accessions. Such diversity is expected to contribute to the overall resilience of the *Citrus* germplasm under environmental change. Based on the epigenetic diversity values, *C. × limon* var. *limon*, *C. × reticulata* and *C. × aurantium* var. *sinensis* can be considered better suited to reflect a broader climate change resilience in terms of their epigenetic profile. In most cases, taxon epigenetic diversity was lower than genetic diversity, while epigenetic and genetic distances were not significantly correlated. The results underscore the significant differences and decoupling between global epigenetic and genetic diversity and expose a complex interplay between epigenetic and genetic factors in the shaping of *Citrus* germplasm diversity.

Patterns of species and within-species orientation in multivariate space were markedly different between genetic and epigenetic data. The orientation in genetic data PCoAs is concordant to the taxa phylogenetic and taxonomic genealogy reflecting the common ancestry of species, hybrids and varieties. These groups are absent in the epigenetic data PCoAs but are noticeably reestablished within taxa when the un-methylated data sets are used.

Overall, this study revealed that the Greek ex situ *Citrus* germplasm collection harbors substantial and taxon-specific epigenetic diversity that is largely decoupled from the genetic structure, highlighting epigenetic variation as an independent and biologically meaningful layer of diversity that may enhance climate-change resilience beyond what is predicted by genetic ancestry and diversity alone. In this respect, future work should concentrate on increasing the numbers of accessions and fitness surrogates studied, and performing high-depth (WGBS) epigenomic analyses.

## Figures and Tables

**Table 1:** Adapters and primers used for the Methylation-Sensitive Amplified Polymorphism (MSAP) analysis of the *Citrus* accessions.

Adapters/Primers	5′ to 3′ Sequence
EcoRI adapter	CTCGTAGACTGCGTACC
	AATTGGTACGCAGTC
EcoRI + 1	GACTGCGTACCAATTC+A
EcoRI + 3	+ACT
	+AAC
	+ATG
	+AAG
HpaII/MspI adapter	GACGATGAGTCTCGAT
	CGATCGAGACTCAT
HpaII/MspI + 1	ATGAGTCTCGATCGG+A
	ATGAGTCTCGATCGG+T
HpaII/MspI + 3	+AAT
	+ACT

**Table 2:** Epigenetic diversity of the Greek national *Citrus* spp. germplasm collection. Analyzed parameters: Number of accessions (N), number of different alleles (*N_a_*), effective number of alleles (*A_e_*), Shannon’s Diversity Index (*I_epi_*), diversity (*h*), unbiased diversity (*uh*), and percentage of polymorphism (*P_epi_*).

Taxa	N	*P_epi_*	*h*	*uh*	*N_a_*	*N_e_*	*I_epi_*
Bergamot	3	24.00	0.11	0.16	0.49	1.19	0.15
Citron	3	22.67	0.10	0.15	0.45	1.18	0.14
Lime	2	18.67	0.09	0.19	0.37	1.18	0.13
Lemon	12	53.33	0.12	0.13	1.07	1.17	0.20
Mandarin	10	46.67	0.12	0.13	0.93	1.18	0.20
Orange	19	77.33	0.14	0.15	1.55	1.19	0.24
*Citrus* spp. mean		40.44	0.11	0.15	0.81	1.18	0.18
*Citrus* spp. total	49						

**Table 3:** Genetic diversity of the Greek national *Citrus* spp. germplasm collection, based on data from Tourvas et al. [26]. Analyzed parameters: Number of accessions (N), number of alleles (*N_a_*), effective number of alleles (*A_e_*), Shannon’s Diversity Index (*I*), observed heterozygosity (*H_o_*), expected heterozygosity (*H_e_*), and percentage of polymorphism (*P%*).

Taxa	N	*N_a_*	*N_e_*	*I*	*H_o_*	*H_e_*	*P%*
Bergamot	3	2.36	2.10	0.71	0.67	0.54	78.57
Citron	3	2.21	1.72	0.56	0.40	0.40	78.57
Lime	2	2.00	1.92	0.63	0.82	0.58	85.71
Lemon	12	1.86	1.67	0.50	0.65	0.36	78.57
Mandarin	10	2.93	1.75	0.65	0.44	0.38	92.86
Orange	19	1.93	1.44	0.35	0.44	0.24	71.43
*Citrus* spp. mean		2.21	1.77	0.57	0.57	0.42	80.95
*Citrus* spp. total	49						

**Table 4:** Pairwise *t*-tests for epigenetic diversity statistics between the taxa *C. × limon* var. *limon* (*lemon*), *C. × reticulata* (mandarin), and *C. × aurantium* var. *sinensis* (orange) (*p*-values for unbiased epigenetic diversity (*h*) above the diagonal; *p*-values for Shannon’s Diversity Index (*I_epi_*) below the diagonal).

Species	*C. × limon* var. *limon*	*C. × reticulata*	*C. × aurantium* var. *sinensis*
***C. × limon* var. *limon***	-	1	0.4049
***C. × reticulata***	0.8453	-	0.4394
***C. × aurantium* var. *sinensis***	0.2299	0.1855	-

**Table 5:** Mantel test results of the correlation between genetic and epigenetic distances, using the global citrus and the different taxa data sets.

Data Sets	R^2^	*p*-Value
Overall (Genetic and Epigenetic Data)	0.0023	0.230
*C. × limon* var. *limon* (lemon)	0.0155	0.360
*C. × reticulata* (mandarin)	0.0542	0.180
*C. × aurantium* var. *sinensis* (orange)	0.0235	0.160

## Data Availability

The DNA methylation (MSAP) data matrix is available from the corresponding author upon request.

## Appendix A

**Table A1:** List of accessions investigated from the *Citrus* Greek National Germplasm Collection.

#	Accessions	Pre-Defined Group	Accession Distribution/Origin
1	Bergamoto Chanion	Bergamot	Greek Landrace
2	Bergamoto Fantastico VCR	Bergamot	Italy
3	Bergamot Femminello	Bergamot	Italy
4	Fymatiodes	Citron	Greek Landrace
5	Cretan Smooth	Citron	Greek Landrace
6	Diamante	Citron	Italy
7	Nouvel Athos	Lemon	Georgia
8	Karistino	Lemon	Greek Landrace
9	Vakalou	Lemon	Greek Landrace
10	Maglino Xylokastrou	Lemon	Greek Landrace
11	Karistini Xylokastrou	Lemon	Greek Landrace
12	Adamopoulou	Lemon	Greek Landrace
13	Lapithou	Lemon	Greek Landrace
14	Zampetaki	Lemon	Greek Landrace
15	Nouvel Athos Porou	Lemon	Greek Landrace
16	Eureka	Lemon	USA
17	Ziagara Bianca	Lemon	Italy
18	Mikrokarpo Messaras	Lemon	Greek Landrace
19	Pastolemono Chiou	Lime	Greek Landrace
20	Mexican Lime	Lime	East Indies
21	Clementine Porou	Mandarin Clementine	Greek Landrace
22	Clementine SRA 63	Mandarin Clementine	Algeria
23	Common Aisinis	Mandarin Common	Greek Landrace
24	Common Chania	Mandarin Common	Greek Landrace
25	Wilking	Mandarin Common	USA
26	Willowleaf	Mandarin Common	Mediterranean Basin
27	Common Chios	Mandarin Common	Greek Landrace
28	Common Chiou mandarin	Mandarin Common	Greek Landrace
29	Common Mesogeiako	Mandarin Common	Mediterranean Basin
30	Nova No 158	Mandarin Hybrid	USA
31	Botsato Artas 1	Orange Common	Greek Landrace
32	Botsato Artas 2	Orange Common	Greek Landrace
33	Thilikoportokalia	Orange Common	Greek Landrace
34	Plake Artas	Orange Common	Greek Landrace
35	Stroggilo Artas	Orange Common	Greek Landrace
36	Aspermo Artas	Orange Common	Greek Landrace
37	Lainato Chanion	Orange Common	Greek Landrace
38	Skliro Timpakiou	Orange Common	Greek Landrace
39	Soultani Cretan	Orange Common	Greek Landrace
40	Jaffa	Orange Common	Israel
41	W. Navel SRA 100	Orange Navel	France
42	Navellina Artas	Orange Navel	Greek Landrace
43	Matsitiko Chiou	Orange Sanguine	Greek Landrace
44	Sanguine Aigiou	Orange Sanguine	Greek Landrace
45	Sanguine Gouritsis	Orange Sanguine	Greek Landrace
46	Moro	Orange Sanguine	Greek Landrace
47	Valencia Oval Porou	Orange Valencia	Greek Landrace
48	Valencia Mesologgiou	Orange Valencia	Greek Landrace
49	Valencia Frost	Orange Valencia	USA
